# Impaired Motor Learning Following a Pain Episode in Intact Rats

**DOI:** 10.3389/fneur.2019.00927

**Published:** 2019-08-27

**Authors:** Maxime Huot-Lavoie, Windsor Kwan-Chun Ting, Maxime Demers, Catherine Mercier, Christian Ethier

**Affiliations:** ^1^CERVO Research Center, Psychiatry and Neurosciences Department, Faculty of Medicine, Université Laval, Quebec City, QC, Canada; ^2^Centre for Interdisciplinary Research in Rehabilitation and Social Integration, Department of Rehabilitation, Faculty of Medicine, Université Laval, Quebec City, QC, Canada

**Keywords:** motor learning, pain, nociception, metaplasticity, plasticity, capsaicin, complete Freund's adjuvant, rehabilitation

## Abstract

Motor learning and pain are important factors influencing rehabilitation. Despite being mostly studied independently from each other, important interactions exist between them in the context of spinal cord injury, whether to the spinal cord or the body. Ongoing or recent past episodes of nociceptive activity can prevent motor learning in spinalized rats. In intact animals, it has been proposed that supraspinal activity could counter the repressive effect of nociception on motor system plasticity, but this has not yet been verified in behavioral conditions. The aim of this study was to test whether a recent episode of nociception affects subsequent motor learning in intact animals. We trained rodents to walk on a custom-made horizontal ladder. After initial training, the rats underwent a week-long rest, during which they were randomly assigned to a control group, or one out of two pain conditions. Nociceptive stimuli of different durations were induced through capsaicin or Complete Freund's Adjuvant injections and timed so that the mechanical hypersensitivity had entirely subsided by the end of the resting period. Training then resumed on a modified version of the horizontal ladder. We evaluated the animals' ability to adapt to the modified task by measuring their transit time and paw misplacements over 4 days. Our results show that prior pain episodes do affect motor learning in neurologically intact rats. Motor learning deficits also seem to be influenced by the duration of the pain episode. Rats receiving a subcutaneous injection of capsaicin displayed immediate signs of mechanical hypersensitivity, which subsided rapidly. Nonetheless, they still showed learning deficits 24 h after injection. Rats who received a Complete Freund's Adjuvant injection displayed mechanical hypersensitivity for up to 7 days during the resting period. When trained on the modified ladder task upon returning to normal sensitivity levels, these rats exhibited more prolonged motor learning deficits, extending over 3 days. Our results suggest that prior pain episodes can negatively influence motor learning, and that the duration of the impairment relates to the duration of the pain episode. Our results highlight the importance of addressing pain together with motor training after injury.

## Introduction

The influence of pain on motor learning is relevant in many contexts, such as rehabilitation after spinal cord injury (SCI) or recovery of athletic sport performance after injury. Almost 80% of people with a SCI will face neuropathic pain, of which more than half will continue to experience chronic pain (1, 2). Professional athletes frequently have to deal with sport injuries and pain, and their significant impact on immediate or long-term motor performance (3).

Motor activity adapts in many ways during acute pain phases, including decrease (4, 5) and increase (6) of voluntary electromyographic activity (7). A recent theory proposes a redistribution of muscle activity in the context of pain in order to reduce the inconvenience induced by movement (8). In addition to the direct modulation of motor activity, acute nociceptive signals may also impair the ability to learn a novel motor task. Nociceptive activity during the acquisition phase of a locomotor task can result in motor learning deficits (9). Capsaicin was also used to demonstrate that intra-oral nociceptive signals can interfere with motor acquisition and associated with modulation of corticospinal excitability in a tongue protraction task in humans (10, 11).

Besides the acute effects of pain on motor activity and plasticity, an increasing body of evidence points to long-term bidirectional interactions between nociception and motor activity. Animal studies have shown that motor activity may decrease the occurrence or intensity of pathological pain after neurological injury (12, 13). Studies in spinally lesioned rats showed that activity in the nociceptive pathways interferes with future neuroplasticity mechanisms involved in motor learning and rehabilitation (14, 15). This interaction between pain and motor learning suggests an important form of metaplasticity. Metaplasticity is “the plasticity of plasticity,” or the idea that a number of factors, including past activity, influences not only the current synaptic strength, but also its propensity for future changes (16). Metaplasticity is characterized by three main criteria: 1- the changes in neuronal activity need to persist beyond the treatment duration, 2- they have to impact the capacity to induce plasticity, not only the responsiveness of the system, and 3- the metaplasticity requires that the effects on the malleability of the nervous system are not due to a permanent alteration (16). In the cases described above, nociception-induced metaplasticity reflects a competition between motor and nociceptive signals to take over control of plasticity in the central nervous system.

Using a validated spinal motor learning paradigm, it has been shown that rats with complete spinal cord transection can learn to maintain their hindlimb in a flexed position to avoid a conditioned nociceptive stimulus (17). This experiment clearly illustrated the possibility of motor learning and plasticity in isolated spinal cord circuits. Using the same paradigm, it was demonstrated more recently that prior pain episodes can reduce the plasticity of spinal cord motor circuits in spinalized rats (18–21). In fact, nociceptive electrical stimulation impairs instrumental learning for 48 h (19). In addition, capsaicin-induced nociceptive activity causes motor learning deficits for at least 24 h after the injection (22). These observations revealed a form of nociception-induced metaplasticity, by which past painful stimuli prevented plasticity in motor circuits of the isolated spinal cord.

The inhibitory effect of pain-induced metaplasticity on motor circuit has mostly been observed in spinally transected rats (14, 21, 23). However, it has been proposed that in intact conditions, descending modulation mechanisms could limit the influence of nociceptive signals on spinal motor learning. Using electrical nociceptive stimuli, Crown and Grau confirmed that brain-dependent processes can counter spinal learning impairment (23). They compared the effect of a nociceptive stimulus applied immediately before or after complete spinal lesion and found spinal motor learning deficits only in animals that received the nociceptive stimulation after lesion. They suspected that supraspinal serotonergic fibers projecting through the dorsolateral funiculus could counteract the negative effect of prior pain episodes on motor system plasticity. Indeed, lesions to the dorsolateral funiculus led to learning impairment in animals with partial transection of their spinal cord (23), but intrathecal injections of serotonin before nociceptive stimulation in completely transected rats restored motor learning (23). The experiments described above suggested that brain-derived serotonergic input could be sufficient to counter the negative effect of pain plasticity on motor circuit malleability. However, this effect was observed in highly constrained conditions, and it is unclear whether these findings apply to intact animal in behavioral context.

Acute pain has been shown to hinder motor learning in neurologically intact humans (9) and animals (14, 15). It was also shown, in isolated spinal cord models, that prior nociceptive episodes could interfere with subsequent motor learning (21, 23). Indeed, a number of studies have shown that inflammatory agents (capsaicin) or electrical nociceptive stimuli could cause some form of metaplasticity, by which nociceptive signals hinders plasticity in the spinal motor circuits (15, 19–21, 23, 24). However, this has been exclusively shown in lesioned models. Consequently, very little is known about the influence of recent pain episodes on motor adaptation and learning in behaving intact animals.

The aim of our study is to clarify the impact of prior pain episodes on motor learning. We designed an experimental paradigm in which we trained rats to cross an horizontal ladder with moveable rungs. After an initial training, we subjected the rats to a pain episode using inflammatory agents, waited for mechanical sensitivity to subside, and then measured their motor learning abilities by evaluating their performance on a modified version of the ladder with larger gaps. Moreover, we tested both capsaicin and complete Freund's adjuvant (CFA) as inflammatory agents, in order to investigate if short term (hours) and long term (~8 days) inflammatory pain could have a differential effect on subsequent motor learning. This design allowed us to conclude that prior pain episodes do affect motor learning in neurologically intact rats, and that learning deficits were influenced by the duration of the pain episode. Our results provide a better understanding of the interaction between past pain episodes and subsequent motor learning, and highlight the importance of considering the negative effect of nociceptive metaplasticity on motor learning to guide rehabilitation programs.

## Materials and Methods

### Summary

**Figure 1:**
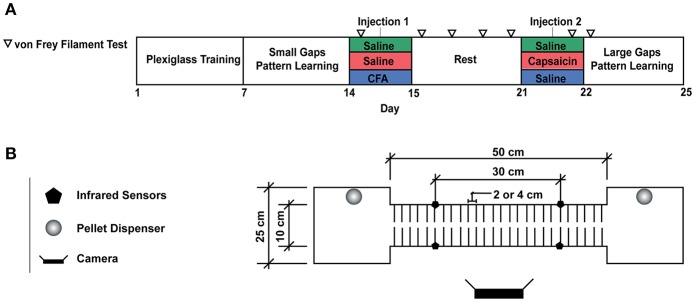
**(A)** Experimental Timeline. **(B)** Motor Learning Task. For the first week of the experiment, animals were trained to run back and forth on the runway to obtain a food reward. A plexiglass sheet was placed on the metal rods to allow rats to become familiar with the task. On the second week of training, the plexiglass floor was removed, and the animals were required to cross the runway by stepping on the metal rods, 2 cm appart. Larger gaps (4 cm between rods) were used to assess motor learning on the fourth week of the experiment. Each rat crossed the runway for a total of 50 times per session and two sessions were completed per day. Transit time was measured by pairs of infrared beam sensors and video recordings were used to detect paw misplacements.

All experiments were approved by Université Laval's Animal Care Committee (CPAUL). We aimed to investigate the effect of a prior pain episode on subsequent motor learning. For this purpose, we trained rats to walk on a custom horizontal ladder, using food rewards sequentially delivered on platforms located at both ends (Figure 1). After an initial training period, the rats underwent a week-long rest period, during which they were randomly assigned to a control group, or one of two pain conditions. Nociceptive stimuli of different durations, mediated by either capsaicin or CFA injections, were timed so that mechanical hypersensitivity had entirely subsided by the end of the resting period (see animals and groups below). Training then resumed on a modified version of the horizontal ladder, where gaps between rungs were made to be twice as wide as in the training session (4 cm instead of 2 cm). We evaluated the animals' ability to learn a new locomotor pattern and adapt to the modified task by measuring their transit time and paw misplacements for two 30-min daily sessions over 4 days and compared these measurements between groups.

### Animals and Groups

Subjects were 19, 200 to 300 g Long Evans male rats. They were individually housed in a temperature-controlled room following a food restriction protocol, with *ad libitum* access to water and maintained on an inverted 12 h light/dark cycle for the full duration of the experiment. Rats were divided into three groups: SAL (saline; *n* = 6), CAP (capsaicin; *n* = 7), and CFA (Complete Freund's Adjuvant; *n* = 6). All rats received two subcutaneous injections on the dorsum of the right hindlimb under isoflurane sedation, on days 15 (injection 1) and 22 (injection 2, Figure 1A). This corresponded respectively to 9 days and 16 h before their first training session in the novel large gap task. SAL rats received 100 uL of 0.9% saline for injection 1 and 50 uL of saline for injection 2. CAP rats received 100 uL of saline for injection 1 and 50 uL of capsaicin 2% (dissolved in a vehicle of 7% Tween 80 and 93% saline 0.9%) for injection 2, therefore timing the onset of the short-duration pain episode 16 h before the first session on the modified horizontal ladder. CFA rats received 100 uL of CFA solution (50 uL of 0.9% saline mixed with 50 uL of 0.25 mg/ml mycobacterium emulsion; Sigma-Aldrich, St. Louis, MO) for injection 1 (9 days before the first large gap session) and 50 uL of saline for injection 2.

This double injection protocol was used instead of single injections in order to standardize the treatment timeline in each group. All rats thus received two injections with the same volume of fluids (100 and 50 uL), 9 days and 16 h, respectively before the first session on the modified horizontal ladder. This ensured that injection volumes or their timing with respect to the motor test could be excluded as influencing factor. Additionally, for the two pain groups (CAP and CFA), the timing of the injections was such that mechanical sensitivity returned to normal shortly before the beginning of the motor learning phase with the larger gaps.

### Automated Horizontal Ladder Task

The animals were trained to a locomotor task, which involved crossing an elevated runway made of metal rods (Figure 1B). Rats were food-restricted 12 h before the initial training began. When rats reached the platforms, located at either end of the runway, a food pellet (Bioserv, Dustless Precision Pellets®, 45 mg) and an audio tone were delivered on the opposite platform in order to reinforce alternate runway crossing to the other side.

As shown in Figure 1A, the total experiment duration was 4 weeks. During the first week of training, the runway was covered with a piece of plexiglass placed on top of the rods to provide a flat and continuous floor. This “plexiglass” training period was performed for 30 min per day over 5 consecutive days. The objective was to familiarize the animals with the task: repeatedly crossing the runway back and forth to obtain food rewards. For the second week of training, the plexiglass was removed, exposing the metal rods positioned 2 cm apart. The rats easily learned to perform this “small gap” task while walking directly on the metal rods. By the end of the week, rats had become proficient as illustrated by stable transit times (Figure 3C). On the third week, the rats entered a rest period, with *ad libitum* access to food and water. Injections were performed at the beginning and end of this period (see animals and groups above), and mechanical sensitivity was assessed throughout (see tactile testing below). At the beginning of the fourth week, the horizontal ladder training resumed, with larger gaps between rods (4 cm between metal rods). We then required all animals to cross the runway for an exact number of 50 times per session.

### Behavioral Measures and Analyses

During the large gaps learning phase, rats underwent two training sessions per day, separated by ~6 h, for 4 consecutive days. We assessed the rats' ability to learn a new locomotor pattern by measuring their transit time and evaluating foot faults. The transit time was obtained using a pair of break beam sensors located 30 cm apart on the runway. Video recordings were used to quantify the number of trials containing paw positioning errors. An evaluator blinded to the animals' treatment group scored the videos.

Rats sometimes stopped on the runway, seemingly to explore the environment. To avoid including outliers in our data, trials during which the rats interrupted their course for reasons other than misplacing their paws on the ladder rungs were excluded from the transit time analyses. Because the number of uninterrupted trials per rat differed for each session, we decided to quantify transit times by including only the first 20 uninterrupted trials for each rat. However, all rats performed exactly 50 trials per session, and all the trials were included in the paw positioning analysis. The data for runway transit times were normalized with respect to each rat's performance over the last two sessions of the small gaps training before the pain episode (Figure 3C).

$$T=(T_{0}-P)*exp(-K*Trials)+P$$

The extra sum-of-squares *F*-test was used to compare different regression models (linear, exponential) and led us to choose the exponential model for both runway transit time and positioning errors. Replication tests also confirmed the adequacy of this model. The non-linear regression fit equation is frequently used in the motor learning literature (25, 26), and in our case serves as a measure of motor performance (transit time, T), which decreases from an initial T_0_ value to an asymptotic plateau (P) by the way of a negative exponential learning rate K (a higher K representing a faster learning rate). We fit the T_0_ and K parameters for each group independently. As the Dunnett's multiple comparisons test following a mixed analysis did not show statistically significant differences in the transit times between groups for the last session of the week (Figure 3B), the final performance parameter (Plateau, P) was fixed at the mean transit time of the two SAL group last sessions, for all the animals (0.678).

Foot misplacements were evaluated from video recordings taken from a sagittal plane relative to the horizontal ladder. A binary notation was used and a score of 0 was assigned to each trial in which the animal made no mispositioning of the paw during an entire crossing. A score of 1 was assigned to any trials containing paw positioning errors (misalignment, slippage). All 50 trials were used for the analysis of positioning errors in each session, regardless of whether or not the animals paused.

### Tactile Testing

**Figure 2:**
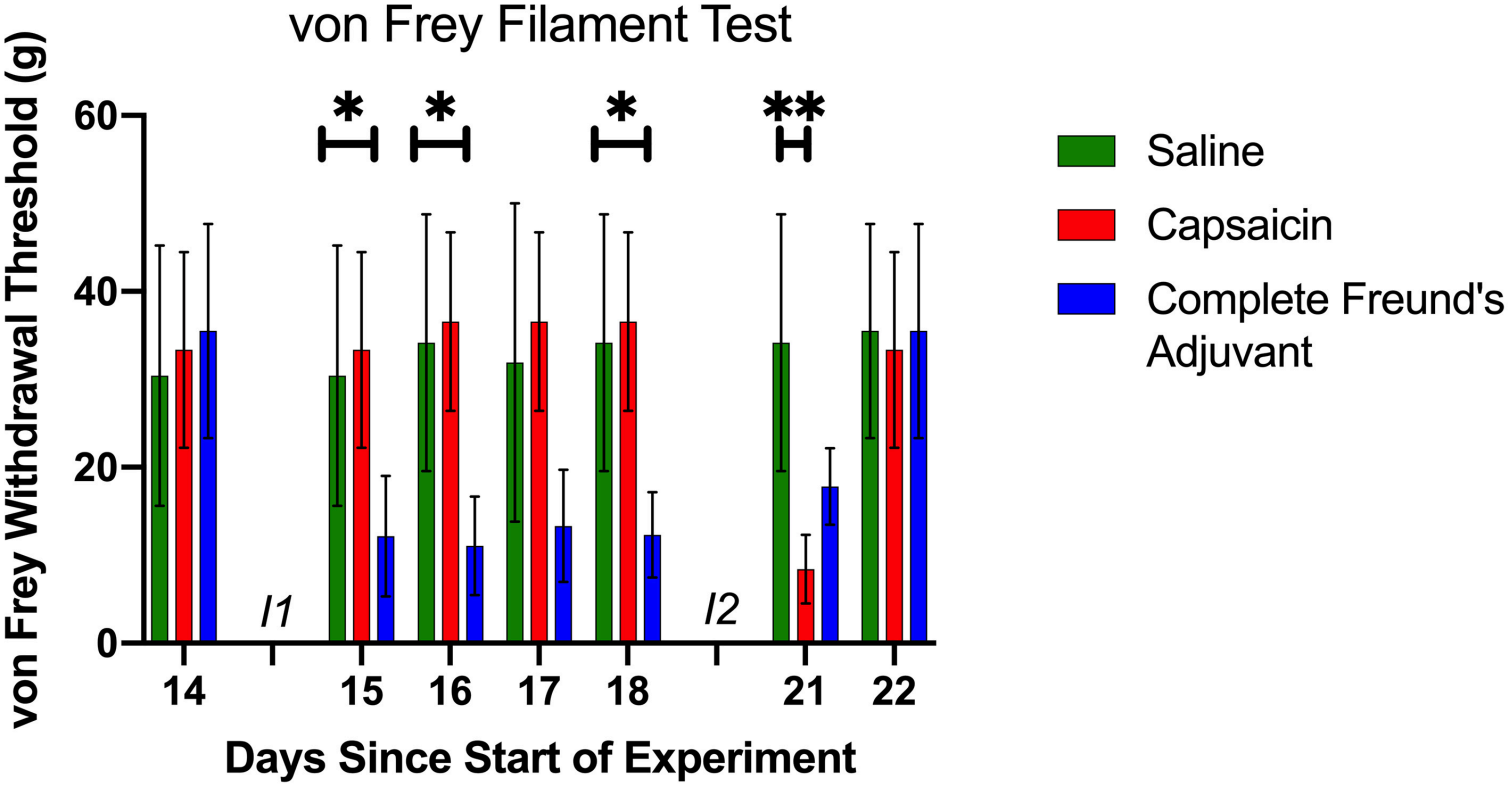
Mechanical Sensitivity. The von Frey filament test was used for a total of seven times prior to the beginning of the motor learning data collection (see also Figure 1A). Baseline data was collected on day 14, before the first injection. At this point, the three groups had a similar paw withdrawal threshold. Following the first injection (I1) (day 15 to day 18), CFA group had a lower threshold than the two other groups. Day 21 data shows that CAP group had a significant lower withdrawal threshold 30 min after injection 2 (I2). Day 22 von Frey filament test shows that all groups had a similar threshold before the beginning of the large gaps pattern learning task, and that the sensitivity had returned to baseline. *p < 0.05 and **p < 0.01.

Mechanical sensitivity is a commonly used measure of allodynia and hyperalgesia in animal and human pain models (27, 28). Von Frey filaments are a well-established method to detect paw withdrawal threshold in rodents (29), which allows for accurate measurement of cutaneous sensitivity associated with several pathological conditions (27, 30). We use a simplified up-down (SUDO) method to measure mechanical sensitivity of the injected paw using Von Frey filament (28). This technique offers an accurate quantification of mechanical nociception in pain models (28, 31). Withdrawal thresholds were measured seven times in total (Figure 2), which ensured we had a measure of the evolution of tactile hyper reactivity and that we could verify return to normal mechanical sensitivity before the onset of the large gap training phase. The observed motor learning deficits were therefore not caused by mechanical hypersensitivity *per se*, but rather by long-term changes induced by the nociceptive activity.

### Statistics

All motor learning data were analyzed using two main statistical procedures. First, the extra sum of squares F test allowed us to test the adequacy of the exponential fit for our data. Replicate tests for lack of fit also confirmed the adequacy of the model. The extra sum of squares *F*-test was also used to compare multiple parameters (K, T0, Plateau) of the exponential fit for the three different conditions. Additionally, two mixed model analysis with transit time and paw positioning errors respectively, were evaluated. In these models, subject was considered as a random factor and treatment (Saline, CAP and CFA) was considered as a fixed factor. *Post hoc* comparisons were made using Dunnett's multiple comparison tests. In all cases, a criterion of *p* < 0.05 was used to determine statistical significance. Data analysis was performed using GraphPad Prism version 8.1.0 for Mac, GraphPad Software, La Jolla California USA.

## Results

We found that motor learning was negatively affected by a prior episode of nociception induced with both capsaicin and CFA. Rats previously subjected to an injection of inflammatory agents were slower to adapt to the novel ladder pattern with larger gaps, even though hindpaw sensitivity was back to normal. They displayed slower runway crossing times and misplaced their feet on the rungs more often. Learning deficits were more prominent for the CFA group, in which the duration of the nociceptive episode was also longer.

### Pain and Mechanical Sensitivity

Capsaicin and CFA injections both induced a predictable drop in paw withdrawal threshold, which lasted several minutes and ~8 days, respectively. The duration of mechanical hypersensitivity was in line with other studies using similar volumes and concentrations (27, 32). Figure 2 shows the evolution of the paw withdrawal threshold for each group throughout the resting period, including the time around both injections. A significant and prolonged decrease in sensitivity threshold was observed for rats subjected to CFA injection. The CAP group exhibited a significant acute decrease in sensitivity threshold only for the tactile assessment occurring 30 min after capsaicin injection. Saline control injections had no observable effect on paw withdrawal thresholds. Withdrawal thresholds were also measured just prior to the onset of the motor task with large gaps. At that point, all three groups displayed a similar sensitivity threshold. We also performed a mixed-effect analysis followed by Tukey's multiple comparison test to verify each group's sensitivity compared to baseline. It showed, as expected from across-groups comparisons (Figure 2), significant decreases in sensitivity threshold relative to baseline for the CFA group from days 15 to 21 inclusively. CAP rats displayed a significant decrease in threshold only for the test performed on day 21. There were no significant changes in sensitivity for the control group on any days. On day 22, immediately before the large gaps test begun, the von Frey fiber test did not show any difference in threshold compare to baseline for any of the groups (Figure 2). In addition, visual inspection did not show any sign of swelling in the injected paw before the beginning of the evaluation. We conclude that the motor learning deficits observed in our experiment were not caused by ongoing nociception *per se*, but rather by changes in the malleability of neuronal circuits induced by a prior pain episode, which reflect a form of metaplasticity.

### Runway Transit Time

**Figure 3:**
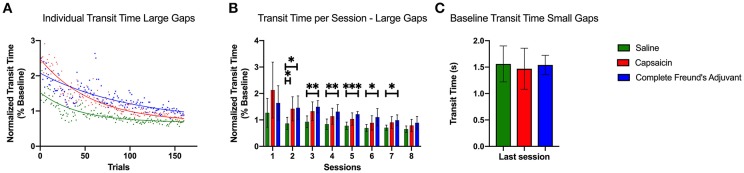
Transit time during large gaps pattern learning task. **(A)** Exponential decay fits of the transit times for the first 20 uninterrupted trials of each session. The trial times are averaged across all rats among each group. All curves were significantly different from each other (*p* < 0.0001). The initial performance (Y_0_, Equation 1), the learning rate (K) and the half-life (number of trials needed to reach 50% of plateau) were found to be significantly different for each condition. **(B)** Bar plot representing the median transit time of all uninterrupted trials for each session (two sessions per day over 4 days). Capsaicin injection induced a significant motor learning deficit 24 h after injection 2 (*p* = 0.0434). The CFA treated group had a prolonged learning deficit that lasted for 72 h. **(C)** Bar plot representing the baseline median transit time for the last session of the small gaps training week. Transit times show no significant differences between the groups. Error bars show the 95% confidence interval. *p < 0.05, **p < 0.01, and ***p < 0.001.

Figure 3 shows transit times for the first 20 uninterrupted runway crossings of each session for rats from the three groups. This data is normalized for each rat to its own average transit time achieved over the last two sessions of the small gaps training. The three groups showed an initial increase in transit time with respect to the small gap performance, followed by a gradual adaptation and increase in performance. By the end of the test week, all the groups had retrieved or surpassed their initial performance. However, rats in the SAL group improved their performance rapidly, whereas CAP and CFA rats took longer to reach the same speed on the new ladder pattern.

We fit exponential decay equations to transit time data pooled for each group (Figure 3A) to compare the three groups' motor performance and learning rates (Equation 1). Extra sum of squares comparisons revealed several differences in performance between groups in the exponential fit model. The SAL group had the lowest value at T_0_ = 1.52 (CAP, T_0_ = 2.47; CFA T_0_ = 2.07). The K parameter, which captured the learning rate, was also significantly different between each group (*p* < 0.0001). It also showed that the SAL group learned significantly faster (K = 0.023, 95% confidence interval (CI) 0.020 to 0.027) than the CAP (K = 0.017 CI 0.016 to 0.020) and the CFA group (*K* = 0.010 CI 0.008 to 0.011). The half-life, representing the number of trials required to reach the middle level of performance between the initial and the final performance levels, also differed for each data set (*p* < 0.0001). The SAL group (half-life = 30.08 trials CI 25.86 to 34.98) was followed by the CAP group (half-life = 39.80 trials CI 35.53 to 44.70) and then by the CFA group (half-life = 71.55 trials CI 61.56 to 84.48). Taken together, these results show that the SAL group learned faster than the pain groups and provide quantitative evidence that the capsaicin-induced learning deficit is smaller than the one evoked by CFA.

Figure 3B shows the median normalized transit times for the first 20 uninterrupted trials for each session. The variability of transit times was very high for the very first session on the large gaps, which prevented statistical tests from confirming any significant differences between groups. However, Dunnet multiple comparisons tests performed on individual sessions show that capsaicin treated rats took more time to cross the runway 24 h after the inflammation agent injection compared with SAL group (*p* = 0.0434). Starting on day 2 (session #3), the difference in transit times between CAP and SAL groups were not significant. On the other hand, except for the first session, the CFA group's performance was significantly lower than the saline treated group for every single session, until the penultimate session. No statistical differences between groups could be observed for the last session. The impact of inflammatory pain on motor learning was thus different depending on the treatment used (CAP or CFA). This suggests that the duration of nociceptive episodes could be a determining factor for learning deficits induced by inflammatory pain.

### Positioning Error

**Figure 4:**
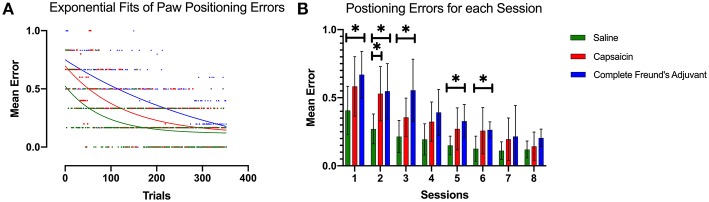
Paw positioning errors. **(A)** Exponential decay fits of paw positioning scores for all trials. The score is averaged across all rats among each group. This model revealed a different curve for each data set (*p* < 0.0001). All curves were significantly different from each other (*p* < 0.0001). The initial performance (Y_0_, Equation 1), the learning rate (K) and the half-life (number of trials needed to reach 50% of plateau) were found to be significantly different for each condition. **(B)** Bar plot representing the mean error in paw positioning. Dunnett's multiple comparisons show that the capsaicin group had a significant learning impairment that was observable 24 h after injection 2 compared with saline (*p* = 0.0370). On the other hand, learning impairment induced by CFA seems to persist longer and is significantly different from the saline group, up to 72 h after the second injection. Error bars show the 95% confidence interval. *p < 0.05.

Figure 4 shows the paw positioning errors that occurred over the 50 runway crossings of each session for each group. This data combines bilateral paw positioning errors identified through video analysis. We found that this measure, which more directly quantifies motor learning and impairment, was significantly dependent on the type of treatment (*p* = 0.0309).

Again, we used the exponential decay equation to fit our data and to examine differences between groups (Figure 4A). The K (half-life) parameter showed that the SAL group improved its performance faster than the other groups (Saline K = 0.012 CI 0.006 to 0.019), and that the CAP group was able to learn faster than CFA group (CAP: K = 0.008 CI 0.005 to 0.011; CFA: K = 0.003 CI 0.00 to 0.006). The significantly different observations from the extra sum of squares *F*-test (*p* = 0.0033) were consistent with the results from runway transit time analysis and suggested that prior pain episodes could interfere with motor learning. The half-life of the SAL group fit (57.67 trials CI 36.34 to 100.7) was shorter than the one of the CAP group (87.25 trials 60.52 to 144.2), and these two were followed by the CFA group (235.20 trials CI 122.1 to 400.0), indicating a longer time to adapt to the new locomotor pattern. The initial rate of foot misplacements also differed significantly between groups (*p* = 0.0017). It was lower for SAL animals (Y_0_ = 0.524 CI 0.446 to 0.615) than for the CAP group (Y_0_ = 0.697 CI 0.624 to 0.776) and the CFA group (Y_0_ = 0.750 CI 0.685 to 0.820).

Mixed effect modeling demonstrated that the results from the mean paw positioning error were consistent with the transit time data described above (Figure 4B). Dunnett's multiple comparison test showed that CAP group had significantly more paw misplacements 24 h after injection (*p* = 0.0374) (session #2), but not for the very first session, possibly again because of large variance. The CFA-induced impairment was observable up to 72 h post injection. The three groups also reached comparable performance after multiple sessions.

## Discussion

Our results show that prior episodes of nociceptive activity induced by inflammatory agents affected motor learning in neurologically intact rats. The motor learning deficits were related to the duration of the nociceptive episodes. These findings demonstrate the importance of considering nociceptive-induced metaplasticity in the context of rehabilitation.

We showed that rats which experienced previous nociceptive episodes learned locomotor tasks significantly slower than the control group. The SAL animals were the fastest learners, followed by the CAP and CFA-treated animals. The mixed model analysis demonstrated a significant difference between each group, for both runway transit time and positioning errors. For the last session, no differences could be detected between groups, suggesting that pain-induced metaplasticity could interfere with motor learning without affecting ultimate performance. The alteration of plasticity induced by nociceptive inputs in the present experiment meet the previously described metaplasticity criteria. First, the motor learning impairment persists even if the mechanical sensitivity has reached baseline values. Second, as motor learning is a consequence of plasticity in the motor system, the prior pain episode does not only affect the responsiveness of the system but also its ability to change. Finally, the alteration in plasticity seems to be reversible considering the fact that each group reach a similar performance at the end of each sessions. Meeting all these criteria, we consider that the motor learning deficit induced by a prior pain episode reflect a form of metaplasticity and not just a form of transient modulation of neuronal activity.

Previous studies have revealed that pain-induced metaplasticity could lead to deficits in spinal motor learning in spinalized preparations (19–21). It has been proposed that brain-derived serotonergic efferent projections could counter this repressive effect in neurologically intact conditions (23). However, our results show that a learning deficit is observable in neurologically intact animals, and suggest that in behaving animals, brain-mediated efferences are not sufficient to counter the repressive effect of recent nociceptive signals on motor learning and plasticity. Our observations do not necessarily contradict previous results observed in isolated spinal cord experiments. Rather, they highlight the fact that nociceptive stimuli could influence the malleability of motor circuits at multiple levels of the neural axis. Indeed, as serotonergic efference do prevent the inhibition of motor plasticity in the spinal cord (23), our results point to metaplastic mechanisms occurring at the supraspinal level. One potential target would be the motor cortex, where evidence suggests a relationship between chronic pain and motor-cortex reorganization (33, 34), and where aberrant metaplasticity has been linked to neurological diseases and psychiatric diseases (35).

The runway transit time analyses indicated that CAP treated rats had a higher transit time than the other groups during the early learning phase, while the positioning errors showed that the CFA group had higher initial performance. Transit times can be influenced by motivational and cognitive factors related to stress. This could be particularly true for the very first session of the CAP rats who had more recently experienced a pain episode. In addition to the novelty of the task, this could explain the larger variance seen in the transit time results and the apparent discrepancy between transit time and positioning errors for the first session. There is also a possibility that the CAP rats still showed an acute sensitivity during the first session that was not reflected in the von Frey tests. The quantification of paw positioning errors represents a more direct assessment of motor learning than transit times, which could be also influence by cognitive factors. The quantification of paw positioning errors may be less affected by cognitive factors and may represent a more direct assessment of motor learning. However, both measures showed similar trends and taken together, they suggest that recent inflammatory pain can lead to motor learning impairment. This confirms the importance of the interference of nociception-induced metaplasticity on learning in the motor system of intact rodents.

Previous evidence of nociception-induced metaplasticity come from studies showing that noxious electrical stimulation prior to denervation in rats (as a model of pre-amputation pain) increases self-mutilation after denervation (thought to reflect the development of neuropathic pain). These experiments suggest that some **sensory** effect of nociceptive stimulation is sustained in the absence of further inputs and clearly outlasts the duration of noxious stimulation (36, 37). To our knowledge, no studies have looked at the duration of previous pain episodes in the context of metaplasticity in the **motor** domain. In fact, the interaction between pain-induced metaplasticity and motor system plasticity have mostly been studied investigating short-term pain conditions in animals (15, 19, 21–23, 38) and humans (39). Although CFA and capsaicin both lead to inflammatory responses, they differ in the details of their mechanisms of action (40). Capsaicin's effects are mainly mediated by TRPv1 fibers, whereas CFA has a more global inflammatory action, recruiting not only C fibers, but also providing a complex set of signals to the immune system (41). Nonetheless, both agents have been repeatedly observed to cause transient increases in mechanical sensitivity and inflammation (27). Therefore, it is possible that the difference in their duration of action be the determining factor for the longer motor learning deficits that we observed in our experiment. The CFA treated group, which was subject to prolonged inflammation, required more trials to reach the same level of performance than the other groups. In fact, the learning deficit was observed up to 72 h after the injection. On the other hand, capsaicin, which generates nociceptive activity for a few hours, leads to a learning deficit that was observable only for 24 h in our experimental paradigm. Without ruling out that differences in mechanisms of action between capsaicin and CFA could explain the differences in motor learning, our results suggest that nociceptive-induced motor learning impairment increases with the duration of the prior pain episode.

This first study shows an effect of nociceptive signals on motor learning, but further investigation will be needed to identify the precise mechanisms mediating that effect. An interesting parallel could be drawn between nociception-induced metaplasticity and the vast body of literature on learned helplessness, where uncontrolled aversive stimuli have been shown to disrupt the future ability to learn escaping similar, but controllable stimuli (42, 43). This work has led to the identification a central role for the inhibitory control of the medial prefrontal cortex over brainstem and limbic structures in mediating this metaplasticity effect (44). Moreover, emerging knowledge in the nociception field has highlighted the importance of glial activation in the multisystemic effect of pain. In has been proposed that activation of glial cells by multiple treatments, including inflammation, can alter learning and memory (45, 46). For instance, memory consolidation, involving neuronal plasticity, is disrupted by increased brain proinflammatory cytokine released by glial cells in response to stressors (47). Activation of glia following an immune response or direct injection of glial activator in several brain regions have led to the idea that glial cells could regulate neuronal plasticity in the central nervous system (45). Even if those observation were not directly related to motor learning, recent studies have shown that astrocytic activity is an important determinant for motor-skill learning (48). Considering that the brain-mediated efference should be sufficient to counter spinal learning deficits in the spinal cord (23), future work should look at the impact of supraspinal glial cells activations as a potential mediator of the motor learning deficit following a nociceptive episode in intact nervous system.

This possibility emphasizes the importance of treating pain rapidly for rehabilitation after neusrological injury. The first three months after a neurological injury such as a stroke or spinal cord injury are determinant for the long-term prospects of recovery (49). Because of this limited “window of opportunity” during which plasticity is enhanced, interference from pain-induced metaplasticity could have lasting consequences for the recovery of motor function. This could also apply to the fields of sports and human performance. Rapid control of pain induced by peripheral injury should be considered to promote an earlier return to initial performance.

## Conclusion

Taken together, our results suggest that motor learning can be detrimentally impacted by prior pain episodes. More specifically, the extent and duration of learning impairment could be related to the duration of prior pain episodes. The clinical corollary would be that prompt control of pain following trauma could lead to faster recovery of motor functions. Importantly however, it has been shown that treatment with systemic morphine can eliminate behavioral signs of pain without necessarily protecting from learning deficits induced by nociceptive inputs (50, 51). A better understanding of how pain-induced metaplasticity interferes with motor learning in neurologically intact systems can guide efforts to develop more effective therapeutic interventions. Research studies too often consider motor recovery and pain as separate issues, whereas there are clearly important interactions between them (52).

## Data Availability

The raw data supporting the conclusions of this manuscript will be made available by the authors, with a reasonable request, to any qualified researcher.

## Author Contributions

MH-L and CE: planned experiments, analyzed data, and wrote the manuscript. MH-L and MD: performed experiments. CM and WT: critical revisions to the manuscript for intellectual content. All authors approved the final version of the paper for submission.

### Conflict of Interest Statement

The authors declare that the research was conducted in the absence of any commercial or financial relationships that could be construed as a potential conflict of interest.
